# A novel nomogram model for differentiating Kawasaki disease from sepsis

**DOI:** 10.1038/s41598-020-70717-4

**Published:** 2020-08-13

**Authors:** Xiao-Ping Liu, Yi-Shuang Huang, Ho-Chang Kuo, Han-Bing Xia, Yi-Sun, Wei-Dong Huang, Xin-Ling Lang, Chun-Yi Liu, Xi Liu

**Affiliations:** 1 The Department of Emergency and Pediatrics, Shenzhen Baoan Women’s and Children’s Hospital, Jinan University, #56, Yulv St., Baoan District, Shenzhen, 518102 Guangdong China; 2 Kawasaki Disease Center and Department of Pediatrics, Kaohsiung Chang Gung Memorial Hospital and College of Medicine, Chang Gung University, #123, Dapei Rd., Niaosong, Kaohsiung, 83301 Taiwan

**Keywords:** Cardiology, Diseases, Medical research

## Abstract

Kawasaki disease (KD) is a form of systemic vasculitis that occurs in children under the age of 5 years old. Due to prolonged fever and elevated inflammatory markers that are found in both KD and sepsis, the treatment approach differs for each. We enrolled a total of 420 children (227 KD and 193 sepsis) in this study. Logistic regression and a nomogram model were used to analyze the laboratory markers. We randomly selected 247 children as the training modeling group and 173 as the validation group. After completing a logistic regression analysis, white blood cell (WBC), anemia, procalcitonin (PCT), C-reactive protein (CRP), albumin, and alanine transaminase (ALT) demonstrated a significant difference in differentiating KD from sepsis. The patients were scored according to the nomogram, and patients with scores greater than 175 were placed in the high-risk KD group. The area under the curve of the receiver operating characteristic curve (ROC curve) of the modeling group was 0.873, sensitivity was 0.893, and specificity was 0.746, and the ROC curve in the validation group was 0.831, sensitivity was 0.709, and specificity was 0.795. A novel nomogram prediction model may help clinicians differentiate KD from sepsis with high accuracy.

## Introduction

Kawasaki disease (KD), also known as cutaneous mucosal lymph node syndrome, is an acute, self-limiting vasculitis that is common in children under the age of 5 years old, with coronary aneurysms occurring in about 25% of untreated cases. KD has also become the main cause of acquired heart disease in children^1,2^. Early and timely diagnosis, as well as effective treatment, can help reduce coronary artery lesions (CAL) and improve intravenous immunoglobulin (IVIG) treatment response^3,4^. However, both the etiology and pathogenesis of KD remain unclear. Incomplete KD and Kawasaki disease shock syndrome (KDSS) are at a high risk of delayed diagnosis and heart complications. In clinical practice, incomplete KD and KDSS are often misdiagnosed as sepsis or septic shock initially, thus increasing the difficulty in making a KD diagnosis, as well as CAL complications^5,6^.

The differential diagnosis of KD and sepsis in the early stage of onset is challenging. Sepsis is a systemic inflammatory response syndrome caused by infection, which is also an important cause of septic shock and multiple organ dysfunction syndromes (MODS)^7^. The main manifestations and laboratory findings of sepsis include fever, elevated white blood cell count (WBC), C-reactive protein (CRP), and procalcitonin (PCT), which are also the manifestations found in KD^8^. Although the early clinical manifestations and laboratory results of sepsis are similar to KD, their clinical treatments differ substantially. Patients with sepsis need timely and appropriate antimicrobial treatment to control and remove the source of infection^9^. In contrast, antibiotics are unnecessary and ineffective for KD patients, while IVIG infusion with follow-up cardiac ultrasonography are the main treatment methods for KD^2^.

Both KD and sepsis are characterized by fever and elevated inflammatory markers in the acute stage, but the treatments are very different. However, from information evident in previous literature reviews, no relevant or single indicator can differentiate KD from sepsis. Therefore, we conducted a retrospective analysis of patients' medical records to set up a single or multiple index equation for clinicians in early KD evaluation to prevent cardiac injury.

## Results

We enrolled a total of 420 children (mean age: 24.4 ± 18.4 months) in this study, including 254 males (60.5%), 166 females (39.5%), 227 KD patients (54.0%), and 193 sepsis patients (46.0%). According to univariate analysis, we observed no statistically significant differences regarding gender, age, and body weight, as shown in Table 1. The KD group demonstrated statistically significant higher levels of ALT, AST, platelets, and erythrocyte sedimentation rates (ESR) than the sepsis group (p < 0.05), while the KD group had significantly lower levels of PCT, hemoglobin, albumin, WBC, and CRP than the sepsis group, as shown in Table 1 (p < 0.05).

**Table 1:** Demographic data of the Kawasaki disease and sepsis patients.

Group	Kawasaki disease (N = 227)	Sepsis (N = 193)	p-value
Male gender (%)	61.6%	59%	0.58
Age (month)	25.2 ± 18.9	23.3 ± 17.85	0.35
Bodyweight (kg)	11.8 ± 3.6	11.3 ± 3.9	0.37
WBC (× 10^9^/L)	14.7 ± 5.7	24.0 ± 11.1	< 0.001
Hemoglobin (g/L)	104.8 ± 10.9	110.8 ± 12.9	0.001
Platelet (× 10^9^/L)	388.0 ± 156.3	338.8 ± 130.3	0.04
Neutrophil (%)	63.9 ± 14.3	69.9 ± 15.6	0.001
ESR (mm/h)	70.1 ± 32.9	60.1 ± 35.4	0.014
CRP (mg/L)	75.3 ± 57.2	98.9 ± 67.9	< 0.001
PCT (ng/L)	0.96 ± 1.3	4.1 ± 4.9	< 0.001
ALB (g/L)	37.2 ± 5.3	39.4 ± 4.1	< 0.001
ALT (U/L)	57.9 ± 105	26.9 ± 44.9	< 0.001
AST (U/L)	70.4 ± 125.5	43.6 ± 30.9	0.02
Sodium (mmol/L)	137.6 ± 3.1	136.9 ± 2.9	0.656

*WBC* white blood cell, *ESR* erythrocyte sedimentation rate, *CRP* C-reactive protein, *PCT* procalcitonin, *ALB* albumin, *ALT* alanine transaminase, *AST* aspartate aminotransferase.

## Selected model factors

Of those patients, 247 (60%) cases were in the modeling group, including 133 cases of KD (53.8%) and 114 cases of sepsis, and 173 (40%) cases were in the validation group, including 94 cases of KD (54.3%) and 79 cases of sepsis. We conducted multivariate logistic regression analysis for the modeling group. Logistic regression analysis was carried out using the statistically significant factors in the univariate analysis as independent variables.

The p-value of the Hosmer and Lemeshow test in this model was 0.32. After the multiple logistic regression analysis of the cut-off values, we found WBC, hemoglobin, PCT, CRP, ALB, and ALT to be the independent risk factors of KD (Table 2).

**Table 2:** Multivariate logistic regression analysis.

Variable	Sig	OR	95% CI	
			Lower	Upper
WBC (≥ 19.71 × 10^9^/L = 0 ; < 19.71 × 10^9^/L = 1)	< 0.001	7.849	3.619	17.023
HGB (no anemia = 0; anemia = 1)	0.02	2.373	1.143	4.927
PCT (> 0.5 ng/mL = 0; ≤ 0.5 ng/mL = 1)	0.001	3.414	1.637	7.119
CRP (≥ 59.11 mg/L = 0; < 59.11 mg/L = 1)	0.006	3.084	1.377	6.907
ALB (≥ 39.35 g/L = 0; < 39.35 g/L = 1)	0.001	3.496	1.631	7.497
ALT (≤ 40 U/L = 0; > 40 U/L = 1)	< 0.001	8.521	3.296	22.026

*OR* odds ratio, *CI* confidence interval, *WBC* white blood cell, *HGB* hemoglobin, *PCT* procalcitonin, *CRP* C-reactive protein, *ALB* albumin, *ALT* alanine transaminase.

### Predictive nomogram to differentiate KD from sepsis

**Figure 1:**
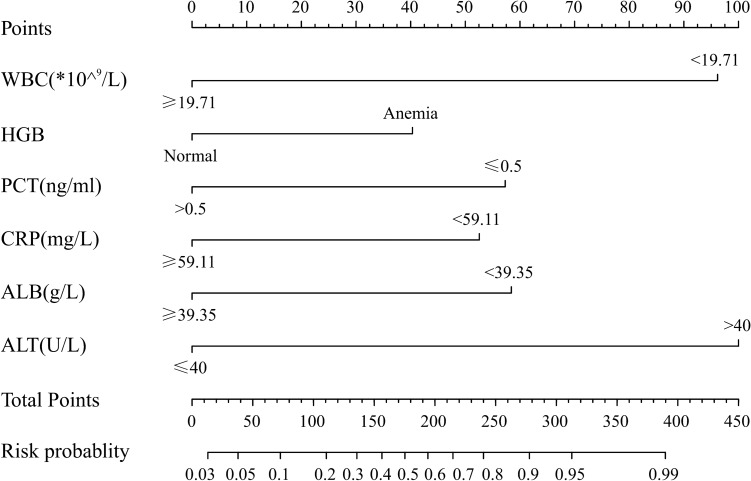
The nomogram prediction score of Kawasaki disease in the differential diagnosis of sepsis. *WBC* white blood cell, *HGB* hemoglobin, *PCT* procalcitonin, *CRP* C-reactive protein, *ALB* albumin, *ALT* alanine transaminase.

According to the results of the multivariate logistics regression analysis, we used WBC, anemia, PCT, CRP, albumin, and ALT to develop the nomogram model (Fig. 1). In the nomogram, ALT was the largest predictor for KD (100 points), followed by WBC (96 points), PCT (57 points), CRP (53 points), and albumin (58 points), while anemia was the smallest predictor (40 points). The total score was 404 points. The incidence of KD corresponding to the score values is shown in Table 3. We use a 50% probability as the classification cutoff value, corresponding to 175 points. In this prediction model, if the patient score is higher than 175 (with a KD probability of more than 50%), while we predict that patient scores of 175 or less may be sepsis, with a probability of more than 50%.

**Table 3:** The incidence risk of Kawasaki disease corresponding to the total score.

Total point	Kawasaki disease risk prediction (%)
13	3
38	5
73	10
111	20
136	30
156	40
175	50
194	60
215	70
240	80
278	90
313	95
390	99

#### Performance of the nomogram

**Figure 2:**
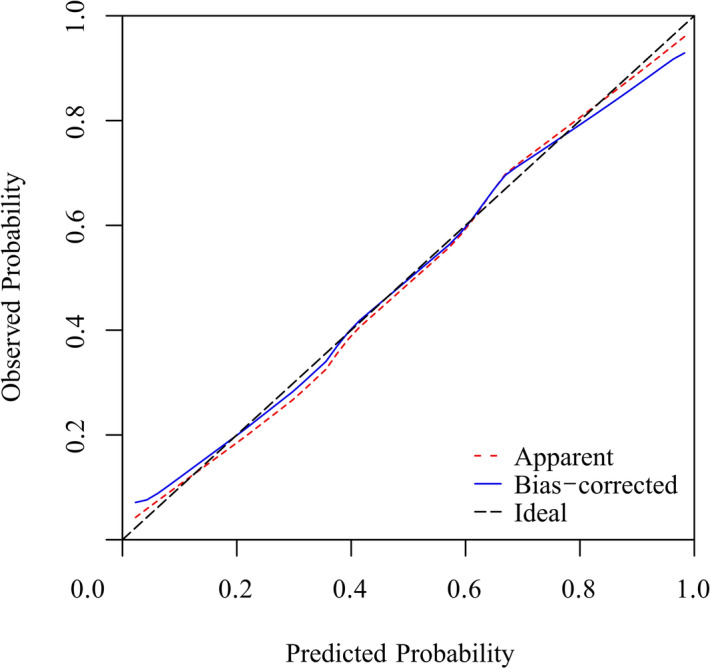
The calibration curves for the nomogram of the modeling group. The x-axis represents the nomogram-predicted probability, and the y-axis represents the actual probability of KD. Perfect prediction would correspond to the 45° dashed black line. The dotted red line represents the entire cohort (n = 247), and the solid blue line is bias-corrected by bootstrapping (B = 1,000 repetitions), indicating the observed nomogram performance.

**Figure 3:**
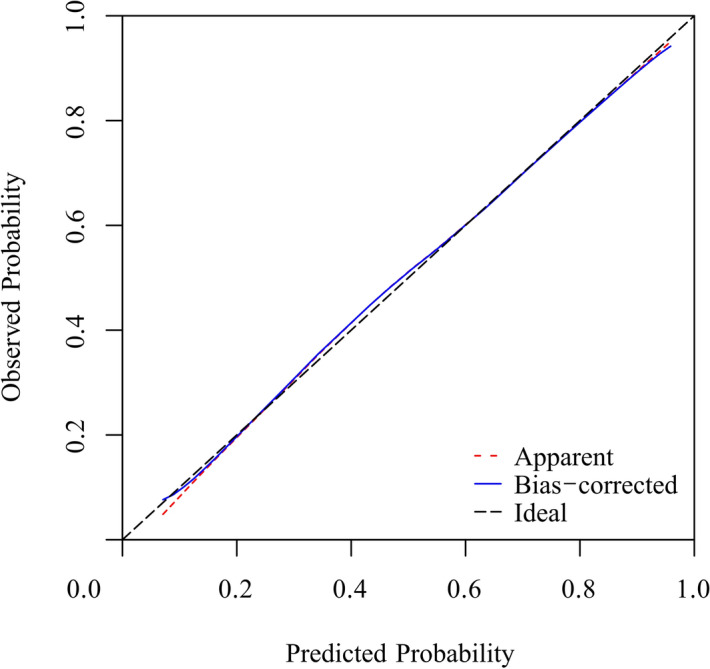
The calibration curves for the nomogram of the validation group. The x-axis represents the nomogram-predicted probability, and the y-axis represents the actual probability of KD. Perfect prediction would correspond to the 45° dashed black line. The dotted red line represents the entire cohort (n = 173), and the solid blue line is bias-corrected by bootstrapping (B = 1,000 repetitions), indicating the observed nomogram performance.

We evaluated performance of the nomogram using discrimination and calibration^10^. Based on the receiver operating characteristic curve (ROC curve) analysis, the nomogram demonstrated good discrimination ability, with an area under the ROC curve of 0.873 (95% confidence interval 0.829–0.918) in the modeling group and 0.831 (95% confidence interval 0.769–0.0.892) in the validation group. The sensitivity and specificity were 0.893 and 0.746, respectively, in the modeling group and 0.709 and 0.795, respectively, in the validation group. Calibration curves of the nomogram for the two groups are shown in Figs. 2 and 3, which demonstrated no apparent fit, with good correspondence between predicted outcome and actual outcome.

## Discussion

KD is currently diagnosed primarily according to clinical manifestations, but no specific laboratory method is available for diagnosis. Furthermore, the diagnosis of incomplete KD is also difficult and increases delayed diagnosis. Although the clinical manifestations and laboratory findings of KD and sepsis differ, some overlap has still been noted. In particular, incomplete KD and KDSS make differentiating the diagnosis from sepsis difficult in clinical practice. The purpose of this study is to compare laboratory findings in KD and sepsis to provide specific characteristics that can help clinicians detect KD earlier.

In this study, compared to the sepsis group, the KD group showed a significant decrease in albumin. Children with KD are often associated with hypoalbuminemia, a pathogenesis that has many explanations. Previous research has reported that the albumin level of KD was significantly lower than that of the febrile control group^11^, indicating that the decrease of albumin may be related to the increase of vascular permeability. Said increase in vascular permeability may be caused by the increased movement of albumin from the blood vessels to the interspaces mediated by hormones, innervations, or cytokines (especially IL-2, interferon-alpha, and IL-6)^12^. Kuo et al. reported that the lower the albumin level, the higher the rate of CAL formation in KD^13^.

Anemia is one of the most common clinical features of KD and is considered to be a marker of the acute phase and disease outcome^14^. In one retrospective study, hemoglobin level was found to be the most important indicator for distinguishing KD from the febrile control group^15^. Furthermore, Lin et al. found that hemoglobin is a useful clinical indicator to distinguish KDSS from toxic shock syndrome (TSS)^4^. Recently, Kuo et al. reported that inflammation-induced down-regulation of hepcidin is associated with anemia in KDSS patients^16^. In our study, the hemoglobin level of the KD patients was significantly lower than that of the sepsis patients. From previous literature reviews, no sepsis-related anemia has yet been reported. Moreover, anemia is a good indicator for distinguishing KD from sepsis and other infectious diseases.

Previous studies have reported that elevated alanine aminotransferase (ALT) is an important laboratory finding for KD, as well as that abnormal liver function including ALT accounts for 37% of total KD children. The elevation of ALT in most children was only slightly elevated, less than twice that of the normal threshold^17–19^. Elevated ALT is also one of the biochemical indicators for predicting IVIG resistance in KD and assisting in incomplete KD diagnosis^2,20^. In our study, the ALT in KD children was significantly higher than that in the sepsis patients and was found to be the most important laboratory indicator (with the highest score of 100) in this novel nomogram model.

Several studies have shown that WBC, CRP, and PCT increased in KD children^21^, and it has been reported that a high CRP level is one of the important laboratory indicators for predicting IVIG resistance in KD^22^. However, CRP, PCT, and other inflammatory indicators are also significantly increased in sepsis patients and have been proposed as potential markers for diagnosing neonatal sepsis^23^. Studies have shown that PCT is significantly higher in KD children than in viral infections and other autoimmune diseases^24^. In our study, the levels of WBC, CRP, and PCT in KD patients were increased but lower than the levels of sepsis patients, indicating that the immune-pathogenesis of KD lies somewhere between sepsis and viral infection. Antibiotics are vital for treating sepsis but may not be as important in the treatment of KD.

According to the literature review, this report is the first to identify KD and sepsis by using ordinary laboratory indicators. Based on clinical laboratory results, we constructed a novel nomogram model with five laboratory markers (WBC, anemia, CRP, PCT, and ALT) for predicting KD and sepsis, which will help clinicians treat KD and sepsis patients more precisely. Furthermore, the timely recognition and treatment of KD can reduce coronary artery damage or progression to KDSS. However, the results of this study still need to be clarified through more cases and from more areas.

Kuo et al.^25^ reported that among the total of 1,065 KD patients, 26 cases admitted to the ICU were identified during a 10-year study period. Shock (73.1%, n = 19) was the most common reason for KD patients to be admitted to the ICU, and ICU KD patients were more likely to receive antibiotics (but not for every KD patient), albumin infusion, and steroids, as well as to require a second dose of IVIG therapy. Kanegaye et al.^26^ reported that patients with KDSS may be resistant to immunoglobulin therapy and require additional anti-inflammatory treatment. In this article, antibiotics were not among the treatment suggestion for KDSS. Altogether, if patients presented with fever and hemodynamic failure with two or three signs suggesting KD, both IVIG and antibiotics were administered in case of KD with uncertain infections, or only IVIG when infections were excluded.

### Conclusion

This is the first study to use a nomogram to develop a novel prediction model that shows that WBC, anemia, PCT, CRP, albumin, and ALT may help clinicians differentiate KD from sepsis with high accuracy.

## Materials and methods

A retrospective analysis was conducted on the medical records of patients who were admitted to the maternal and child health hospital of the Baoan District in Shenzhen, China from 2017 to 2019. All the children were younger than 18 years of age. KD diagnoses were made according to the American Heart Association (AHA) criteria and showed at least four of the following five clinical manifestations: bulbous conjunctival congestion, lips or oral cavity changes, hand and foot symptoms, skin manifestations, and cervical lymph node enlargement^2,27^. Incomplete or atypical KD (N = 47) diagnosis was made in patients with a history of fever lasting more than 5 days but fewer than four of the five major KD clinical diagnostic criteria, with echocardiography showing evidence of coronary artery changes^28^. We compared KD (including typical KD, atypical KD, and Kawasaki disease shock syndrome (KDSS)) with sepsis but not septic shock. Our hospital only had a few cases of KDSS (fewer than 10 cases) during the study period of our recent publication^29^. Sepsis is defined according to the “2016 Surviving Sepsis Campaign”^30^.

The laboratory data of the patients enrolled in this study, including complete blood count/differential count (CBC/DC), C-reactive protein (CRP), aspartate aminotransferase (AST), alanine transaminase (ALT), albumin, procalcitonin (PCT), sodium, and erythrocyte sedimentation rate (ESR), were all collected prior to IVIG treatment in KD patients and before antibiotics were administered to the sepsis patients during admission. The anemia criteria were defined by age according to the World Health Organization (WHO)^31^.

### Statistics

The data in this study were analyzed with mean ± standard deviation (x ± s) used for measurement data and n and percentage used for enumeration data. We compared the normal distribution data using independent sample t-test or single factor analysis of variance. We adopted rank sum test to compare non-normal distribution data. The Chi-square test was used for counter measurement. A p-value less than 0.05 was considered statistically significant. Multivariate logistic regression was applied to analyze the influencing factors of KD and sepsis. Some continuous data were converted into classified data (WBC, neutrophil percentage, CRP, albumin, and ESR), according to the cutoff value when the area of the ROC curve is the maximum value. In part, according to the reference range of the normal values, the upper-limit levels as the cutoff point were converted into classified data (platelet, PCT, ALT, AST). We developed the nomogram model using the result of the multivariate logistics regression model to show the odds ratio and β-factor of each predictor. We adopted Hosmer and Lemeshow to test whether the logistic prediction equation was suitable. The performance of the nomogram was evaluated as reported by Harrell et al.^32^. Furthermore, the validation group was used to verify the scoring results of the nomogram, draw the ROC curve, and calculate the area under the curve. The highest point of the Youden index was used to calculate the sensitivity and specificity of the verification group. The calibration of the models can be assessed using calibration plots, which can predict probabilities against the actual observed risk^10^. SPSS 13.0 statistical software was used for the analysis, and the nomogram was drawn based on the R software (Math Soft, Cambridge, Massachusetts).

### Ethics approval and consent to participate

This study was conducted in accordance with the Declaration of Helsinki. The Institutional Review Board of Baoan Maternal and Child Health Hospital, Shenzhen, China approved the study (IRB No. LLSCHY2019-07-01-02).

### Patient consent

This retrospective data analysis was approved by the Institutional Review Board of Baoan Maternal and Child Health Hospital (IRB No. LLSCHY2019-07-01-02). The need of informed consent in this study was waived.

## Data Availability

The datasets used and/or analyzed during the current study are available from the corresponding author upon reasonable request.
